# Long-Term Care Facilities and Nursing Homes during the First Wave of the COVID-19 Pandemic: A Scoping Review of the Perspectives of Professionals, Families and Residents

**DOI:** 10.3390/ijerph181910099

**Published:** 2021-09-26

**Authors:** Domingo Palacios-Ceña, Rosario Fernández-Peña, Angela Ortega-López, Ana Fernández-Feito, Oscar Bautista-Villaécija, Olga Rodrigo-Pedrosa, José Arnau-Sánchez, Ángel Lizcano-Álvarez

**Affiliations:** 1 Humanities and Qualitative Research in Health Science Research Group, Universidad Rey Juan Carlos (Hum&QRinHS), 28922 Alcorcón, Spain; domingo.palacios@urjc.es; 2 Department of Physical Therapy, Occupational Therapy, Physical Medicine, and Rehabilitation, Universidad Rey Juan Carlos, 28922 Alcorcón, Spain; 3 Faculty of Nursing, Universidad de Cantabria, 39008 Santander, Spain; roser.fernandez@unican.es; 4 Nursing Research Group, IDIVAL, 39011 Santander, Spain; 5 SALBIS Research Group, University of León, 24071 León, Spain; 6 Hospital Universitario Virgen de la Victoria de Málaga, 29010 Málaga, Spain; angelaol73@gmail.com; 7 Instituto de Investigación Biomédica de Málaga-IBIMA, 29010 Málaga, Spain; 8 Department of Medicine, Faculty of Medicine and Health Sciences, Universidad de Oviedo, 33006 Oviedo, Spain; 9 Nursing Research Group, Health Research Institute of Asturias (ISPA), 33011 Oviedo, Spain; 10 Campus Docent Sant Joan de Déu, 08034 Barcelona, Spain; obautista@santjoandedeu.edu.es; 11 School of Nursing of the Sea, Universidad Pompeu Fabra, 08002 Barcelona, Spain; orodrigo@psmar.cat; 12 Directorate General of Health Planning, Research, Pharmacy and Citizen Services, Murcia Region Health Counseling, 30071 Murcia, Spain; jarnau@um.es; 13 Department of Nursing and Stomatology, Universidad Rey Juan Carlos, 28922 Alcorcón, Spain; angel.lizcano@urjc.es

**Keywords:** elderly people over 65, COVID-19, mixed methods, nursing homes, qualitative research, scoping review

## Abstract

The COVID-19 pandemic has had significant repercussions for nursing home residents, their families, and professionals. The objective was to describe the perspectives of residents, their families, and nursing home employees during the COVID-19 pandemic. A scoping review was carried out using the PRISMA Extension for Scoping Reviews. The inclusion criteria were: qualitative and/or mixed methods studies in English, French, Portuguese, and Spanish. The review covers studies published from 11 March 2020 to 15 February 2021. CINAHL, PubMed, Web of Science, ScienceDirect, Scopus, British Nursing Index, Proquest, PsycInfo, and Google Scholar databases were used. We conducted a systematic narrative synthesis, presenting the results narratively and showing descriptive statistics on the studies reviewed. Sixteen documents were obtained from 175 results. Two studies focused on residents and one on their families. The remaining studies looked at professionals. Nursing homes had great difficulty managing resources, which was exacerbated by emotional exhaustion among residents, employees, and family members. In nursing homes, creative initiatives and new forms of leadership appeared to meet emerging needs during the pandemic. The results of the study show the impact of the pandemic on nursing homes and the response capacity present among residents, family members, and professionals.

## 1. Introduction

The COVID-19 pandemic is a global challenge, which has had a direct impact on health systems [1] and economic, social, and legal repercussions [2]. Elderly people have been particularly vulnerable, displaying a greater incidence of infections, adverse outcomes, and mortality [2,3,4] in a similar pattern to other coronavirus outbreaks such as SARS and MERS, which were more lethal to the elderly [5]. The risk of hospitalisation, ICU admission, and mechanical ventilation also increases with age, particularly among people aged over 85 [6].

Elderly people in nursing homes have been one of the worst affected groups in terms of numbers of infections and deaths [7]. In 2020, around half of all COVID-19 deaths occurred in nursing homes [8]. During the pandemic, residents of these facilities experienced a higher risk of infection and a greater impact of the virus due to their fragility, advanced age, and comorbidity, as well as the absence of personal protective equipment, staff shortages, and a lack of rapid diagnostic tests [7]. The conditions in nursing homes and long-term care facilities affected residents, their relatives, and health and social care workers [9]. Among the residents, the pandemic led to reduced activity levels, wellbeing, and cognitive function, and poorer sleep quality [10]. Their physical and emotional health worsened, with rising levels of depression, weight loss, incontinence [11], and loneliness, and an exacerbation of mood and behavioural disorders [12]. The restrictions adopted in response to the pandemic included limiting visits to nursing homes, giving rise to sadness, fear, and concern among family members for the impact of loneliness on residents [13]. Existing studies [14,15] note that nursing home employees experienced increased workload, contradictory instructions and guidelines, communication issues, emotional overload, and fear of contagion [14], alongside high levels of stress due to the social pressure of their work, their exposure to suffering and death, and shortages of staff and personal protective equipment [15].

Several qualitative studies have addressed experiences of the pandemic, exploring the perceptions of hospitalised patients [16], their family members [17], and frontline healthcare workers at hospitals [18,19,20,21]. However, we were unable to find studies that collected and comprehensively examined the perspectives of older persons living in nursing homes and long-term care facilities, employees of these facilities, or family members of residents during the COVID-19 pandemic.

By exploring and describing their experiences, interventions to tackle COVID-19 in nursing homes and long-term care facilities may be adapted to reflect the differences in their functioning and resource capacity when compared with hospitals. Research in this area also represents an opportunity to reveal a lesser-known side of the pandemic, which has been portrayed negatively by some media outlets [14]. The research question underpinning this study was: what published evidence is available on the experiences and perspectives of elderly people, their family members, and/or health and social care workers living in nursing homes and long-term care facilities during the COVID-19 pandemic?

The objective of this scoping review was to describe the experiences and perspectives of residents, family members, and health and social care workers in nursing homes during the COVID-19 pandemic.

## 2. Materials and Methods

### 2.1. Design

The basis for this study was a scoping review. Scoping reviews are a method for summarising knowledge which follow a systematic approach to map evidence on a topic, identify the main concepts, theories, and sources, and determine any gaps in knowledge [22,23]. They may examine the extent (i.e., size), range (i.e., variety), and nature (i.e., characteristics) of the evidence on a particular topic, summarise findings from a body of knowledge that is heterogeneous in terms of methods or discipline, or identify gaps in the literature to aid the planning of future research [22,23]. Our scoping review used the PRISMA Extension for Scoping Reviews (PRISMA-ScR) [23]. PRISMA-ScR was adapted from the original PRISMA Statement and adheres to JBI guidance for scoping reviews [24] and the methodological framework developed by Arksey and O’Malley and refined by Levac [25].

### 2.2. Inclusion and Exclusion Criteria

The study inclusion criteria were original qualitative and/or mixed methods studies written in English, Portuguese, French, and/or Spanish and focusing on the perspectives and experiences of elderly people living in nursing homes and/or long-term care facilities, their families, and health and social workers at these facilities during the COVID-19 pandemic. The review includes studies published between 11 March 2020 (WHO declaration of the COVID-19 pandemic) [26] and 15 February 2021.

The primary qualitative research studies reviewed drew on phenomenology, narrative stories, grounded theory, action research, ethnography, and qualitative case studies, among other methods [27]. A synthesis of qualitative evidence was used to confirm, supplement, and expand on the results [28,29]. The primary mixed methods research studies consulted [30] had to include a qualitative phase in their design, regardless of their structure (sequential, parallel, embedded). Studies based on questionnaires with open-ended questions aiming to explore participants’ perspectives qualitatively were also considered. Particularly, due to the impact of the COVID-19 pandemic on people’s experiences, the study also included first-person testimonials from residents, family members, and health and social care workers.

Residents were defined as people aged over 65 who live in long-term care facilities and nursing homes, regardless of their reason for doing so. Family members were also included, regardless of their degree of kinship. It was also decided to include a wide range of health and social care professionals, regardless of their specific discipline or area.

Study settings were limited to nursing homes and long-term care facilities. For the purposes of this study, nursing homes and long-term care facilities are defined as facilities where elderly people with differing degrees of dependency live permanently or temporarily. These facilities offer health services (nursing, medicine, physiotherapy, occupational therapy, psychology), personal services (hospitality, laundry, cleaning), and social services (social worker). They also offer supervision and assistance with activities of daily living when residents’ physical or mental condition demands care and services beyond accommodation and board. Medical and nursing services are available when required. Skilled nursing care and rehabilitation services for residents delivered on a daily basis were also covered by the study.

Studies were excluded if they: (a) focused on elderly people requiring acute or hospital care, or facilities providing nursing supervision and medical care to elderly persons requiring hospitalisation; (b) were based on epidemiological studies, meta-analysis, or protocols; or (c) described the perspectives of professionals from primary healthcare, hospitals, or other health and social care providers. Policy briefs, books, book chapters, commentaries, and published or unpublished reports from governments and other agencies were also excluded. Internet searches for doctoral theses were not performed due to the short time frame for the study.

### 2.3. Identification of Studies: Search Strategy and Terms

We began with the following databases: CINAHL, PubMed, Web of Science, ScienceDirect, Scopus, British Nursing Index, Proquest, and PsycInfo. Databases and resources containing publications in Spanish were also reviewed, such as SciELO, IBECS, Dialnet, Lilacs-Bireme, Index Foundation, and the Andalusian Health System Virtual Library (BVSSPA). Google Scholar and a manual search focusing on specialised journals in geriatrics and gerontology published by Spanish scientific societies produced additional sources.

A matrix of search terms was drawn up and four search groups obtained. For the search, the authors created combinations with Boolean operators AND and/or OR and the truncation symbol (*). International databases using Spanish as their working language (Dialnet, Lilacs-Bireme, IBECS, Scielo, Index Foundation) were also included. For the searches in Spanish, terms from the thesaurus for each database or free-text terms adapted from the keywords in English were used. The DeCS (Health Sciences Descriptors) from the Virtual Health Library (https://decs.bvsalud.org/E/homepagee.htm, accessed on 8 April 2021) were also included (Table 1). The search was performed using English and Spanish terms. In the event of detecting articles with the abstract in English but available in full text in Portuguese and/or French, their inclusion was agreed upon.

### 2.4. Screening for Eligibility

**Figure 1:**
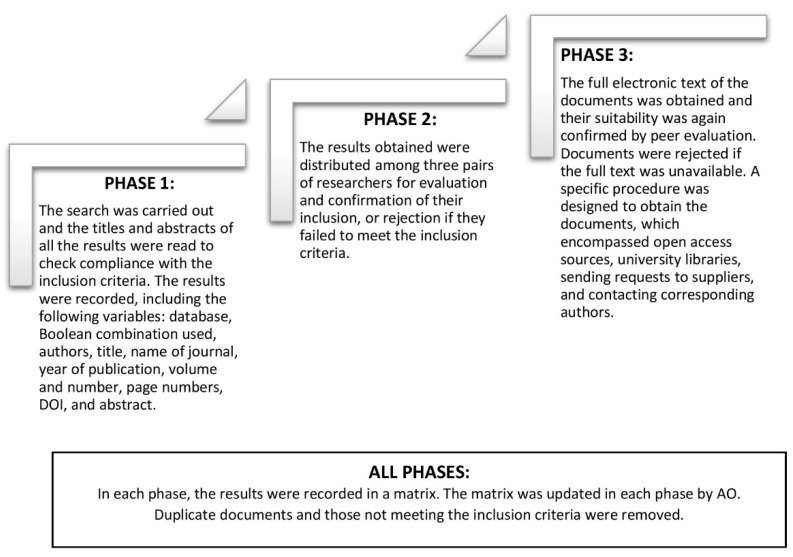
Screening and eligibility phases.

No assessment of study quality was made, as the purpose of this scoping review was to synthesise and describe coverage of the evidence. Before the start of the review, a unified protocol comprising several different phases was elaborated and applied during the search, which was conducted by six researchers (Figure 1). This protocol was decided by consensus among the members of the research team with experience in conducting reviews (DPC, RFP), scoping reviews (AFF), and meta-syntheses (DPC, RFP). At the conclusion of each phase, a telematic meeting of the entire team was held to discuss the incidents and confirm the results obtained.

### 2.5. Data Extraction and Inclusion

Three pairs of researchers were established for the data extraction process (AFF and RFP, DPC and ALA, and AOL and OBV). Group sessions were held to describe and confirm the data obtained. In the event of any hesitation, a consensus decision was reached. The aim of this scoping review was to synthesise and describe coverage of the evidence.

New variables were extracted to explore the characteristics of the selected studies in greater depth (Table 2).

### 2.6. Data Synthesis

Once the data were extracted, we inductively developed a health and social care framework based on the experiences and perspectives of residents, families, and long-term care facility employees during the COVID-19 pandemic. The analysis synthesised the evidence base and identified knowledge gaps regarding health care and the impacts of the pandemic on elderly people, families, and employees.

A systematic narrative synthesis was conducted, with results presented narratively and organised thematically alongside tables showing descriptive statistics on the studies reviewed and their outcomes [31]. This systematic review method involves categorising and re-categorising the findings of two or more studies to produce synthesised findings [24]. Firstly, the studies were closely read and re-read at least twice by three researchers (AOL, OBV, and ALA) to obtain a preliminary understanding. The findings or groups of findings were then extracted. Subsequently, the degree of congruency between the findings was assessed independently by four researchers to ascertain the credibility of the researcher’s interpretations. Thus, similarities and contradictions were sought between the findings, before creating categories. These categories were then repeatedly read and re-read to identify similarities and produce a synthesis of results.

## 3. Results

**Figure 2:**
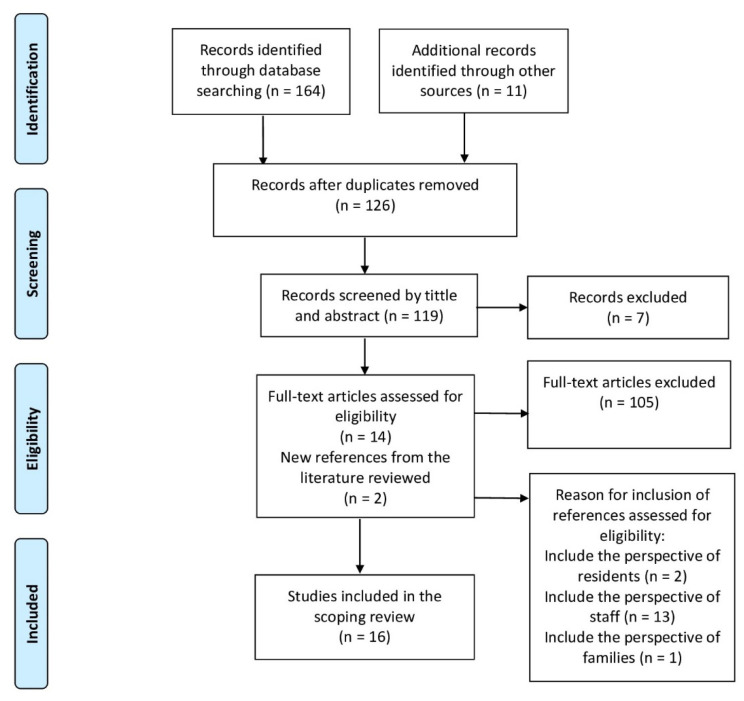
Flow diagram for the scoping review process.

The first search phase produced 175 results, leading to a total of 126 results once duplicates had been removed (*n* = 49). The titles and abstracts were then reviewed to exclude any that did not meet the inclusion criteria. This resulted in a total of 14 studies, which were supplemented by 2 studies obtained from the reference lists of the selected studies. The 16 studies selected are shown in a flowchart of search results (Figure 2) in accordance with the PRISMA-ScR recommendations [22,23].

### 3.1. Study Characteristics

Of the 16 studies included, 2 were conducted in the USA [32,33], 3 in the United Kingdom [34,35,36], 1 in Australia [37], 1 in Germany [38], 1 in Canada [39], 1 in Sweden [40], 1 in Spain [41], 3 in the Netherlands [42,43,44], 1 in Slovenia [45], 1 in Malaysia [46], and 1 in several Latin American (Peru, Mexico) and European (Spain and Italy) countries [47]. Fifteen were in English and only one was in Spanish [41].

With regard to methodology, the 16 studies included 5 mixed methods studies [38,39,42,43,45], 7 qualitative studies [32,34,35,37,41,46,47], 1 short report [36], 1 rapid scoping review [44], and 2 studies using another approach such as a letter or report containing a first-hand testimony [33,40].

A total of 2,262 participants were included: 352 came from qualitative studies and from the qualitative phase of mixed methods studies. Only two studies focused on residents [33,46] and one on family members [38]. The remaining studies analysed professionals. See Supplementary Materials, Table S1.

The synthesis of results produced two main themes: (a) deterioration of the organisational climate, with two sub-themes: “organisational difficulties in responding to the new circumstances” and “experiences of emotional exhaustion and negative perceptions”, and (b) adapting to adversity, with two sub-themes: “innovation and creativity in responses to adversity” and “acknowledging and adapting to shortcomings” (Table 3).

### 3.2. Deterioration of the Organisational Climate

The results obtained show that nursing homes had difficulty with organising and managing material and human resources to respond to the pandemic [32,34,35,36,37,38,39,40,41,42,43,45,47], as well as revealing the emotional exhaustion experienced among residents, employees, and family members [32,33,34,35,38,39,40,41,42,44,45,46,47].

#### 3.2.1. Organisational Difficulties in Responding to the New Circumstances

During the early weeks of the pandemic, there were shortages of PPE and cleaning products (disinfectant, bin liners, etc.). To prevent contagion, employees had to minimise personal contact with residents, which had a negative impact on their care [32,34,40,41,43,47]. The identification of clean and contaminated areas was carried out in a makeshift manner due to a lack of clear guidelines from managers [32,40]. This reorganisation required nursing homes to make decisions on the safety of residents and employees, including protocols for isolation and evacuation or for screening people with symptoms or a positive COVID-19 diagnosis, which increased stress levels [32,38]. This led to errors in counting and recording the numbers of infected and deceased residents [34]. In addition, residents’ physical and social activities were cut back [38,42]. Visits were limited and exhaustively recorded, while families were subjected to preventive hygiene measures such as compulsory PPE and authorisation of only one relative or main carer to visit residents [32]. Despite this, family visits to residents receiving end-of-life care (compassionate care visits) continued [32,41,42].

Meanwhile, the rising numbers of staff taking sick leave exacerbated staff shortages in health and social care facilities [35,39,40,47]. This increased the workload for the remaining staff, with long, exhausting shifts and affected the care of vulnerable elderly people with complex needs [41,45]. Several studies [34,39,45] have linked these conditions to higher fatality rates in the first few weeks of the pandemic. The time available for carers to help residents contact their families via telephone calls or electronic devices was also drastically reduced [37]. The lack of support for health and social care facilities from hospitals led to insufficient availability of medical care, resulting in residents being discharged from hospital early, especially at weekends [34,38]. Nurses were forced to respond to the emerging needs as best they could [38,45], as well as resolving conflicts with family members [41] and other professionals [40].

Finally, the absence of strategic decision-making by the health authorities regarding health and social care facilities impacted nursing homes’ ability to organise, leading to shortcomings in the distribution of PPE supplies and economic resources, and an absence of common, standard rules [34,35,39,40]. Nursing home employees and managers often felt undervalued and abandoned by the public authorities [34,35].

#### 3.2.2. Experiences of Emotional Exhaustion and Negative Perceptions

Emotional exhaustion was a common theme in the studies reviewed, as well as uncertainty about the future, fear of contagion, and bewilderment at the unfolding situation. This was accompanied by health problems in residents and employees, such as sleep disturbances, loss of appetite, lack of mobility, etc. [34,35,40,42,46,47].

One of the causes of emotional exhaustion among elderly people and their carers was loneliness due to a lack of contact with family members, isolation of residents in their rooms, and a sense of having been abandoned by the authorities [40,47]. Families felt that their elderly relatives had been ‘imprisoned’ due to the visiting restrictions [38,40,41,42]. Tensions within families remained even once the restrictions had been relaxed [41].

Changing routines or the loss of these routines (reduction in scheduled activities, changing protocols, suspended visits) and increased emotional distress within nursing homes [35,38,41,47] gave rise to conflicts between staff, residents, and nursing home managers [41]. One of the causes of emotional exhaustion was the handling of end-of-life care, as medical, social, and spiritual care was in short supply and sometimes considered low priority [44]. Managing deaths and funerals was another source of emotional exhaustion because of the impact of the pandemic on administrative procedures (preparing the body, notifying relatives, issuing certificates, obtaining transport, etc.) [32,33,35,40,41,47]. Some studies also highlight difficulties in providing care for vulnerable residents with emotional and cognitive disturbances or mobility issues [34,42] and for those requiring psychotropic medication [44].

Staff recounted their constant struggles and exhaustion due to their excessive workload [35,40] and their emotional fatigue as they became the residents’ ‘substitute family’. In some cases, families put pressure on staff to allow them to contact residents via video call, email, etc. [33].

Professionals also described their growing fear of infecting their own families [38,39,40,47], as well as the stigmatisation and undervaluation of their work by society and the authorities [41,47]. Contradictory information in the media contributed to increasing professional and personal dissatisfaction, requiring psychological attention in some cases [40,41,42,47].

### 3.3. Adapting to Adversity

Despite the adverse conditions inside nursing homes [32,33,34,35,36,37,38,39,40,41,42,43,44,45,46,47], interpersonal relationships were strengthened by the pandemic. Creative initiatives and new forms of leadership emerged to meet arising needs despite administrative difficulties and shortfalls in resources [35].

#### 3.3.1. Innovation and Creativity in Response to Adversity

In response to the pandemic, employees took proactive action to create their own plans to tackle the first wave and lockdown [34,39]. This led to greater group cohesion and communication among nursing home managers, employees, and residents through in-person meetings, video calls, social networks such as WhatsApp™, etc. [34,39,47]. To compensate for the suspension of group activities, staff sought to deliver more personalised, continuous care and support to residents [38,42].

The pandemic acted as a catalyst for innovation among nursing home staff, leading to improved care quality [34] and stronger bonds [41,45]. Strategies were implemented to allow communication with family members (video calls, social media, creation of ‘safe’ areas for visits [39,42,44]. Sometimes, family members were allowed to move into homes to avoid loneliness and isolation, especially in end-of-life situations [34]. Closer relations and greater communication with local communities (primary healthcare facilities, schools, churches, neighbours, independent healthcare organisations, volunteers) were also forged [34,35,37,44]. This led to donations of equipment for leisure and communication (tablets, craft materials, paints, etc.) [34,35,37,44] and guidance from local healthcare professionals [40]. It is important to highlight the work of volunteers, who contacted residents from outside the nursing homes through a variety of channels (telephone, letters, email, etc.) to offer them friendship and support [44].

#### 3.3.2. Acknowledging and Adapting to Shortcomings

One of the most relevant themes was a sense of duty and professional dedication when faced with difficult circumstances [42,47]. Multidisciplinary work at the nursing homes made professionals proud to belong and enhanced group cohesion [35,40,42,45]. Proving themselves capable of adapting also made staff feel more positive, satisfied, and confident [38,40,41,47]. Their professional activity was guided by values such as charity, prompting them to make sacrifices such as working without PPE [34] and staying at their posts for long hours [40]. The professionals displayed their most humane, charitable side during the pandemic. Teams came together to help each other overcome their fears and seek their own mechanisms to build resilience in their professional, personal, and family lives [36,41]. Several studies suggest that adaptation in nursing homes led to greater recognition among the public authorities of the need to support and reorganise these facilities [40,47].

## 4. Discussion

The COVID-19 pandemic hit nursing homes hard, giving rise to organisational difficulties and emotional exhaustion among professionals, residents, and family members, who nonetheless found creative solutions to adapt and bring about change.

Our results show that few studies have explored the perspectives of residents and their families [33,38,43], perhaps due to the restrictions limiting access to nursing homes for research purposes. Meanwhile, the use of digital platforms as tools for data collection (interviews) may have been hindered by a lack of familiarity with this technology among residents and a shortage of devices or resources (internet connection) [48]. Most of the literature focuses on professionals and on the impacts of the pandemic on their organisation and their relationships with residents and their families [32,34,35,36,37,39,40,41,42,43,44,45,47]. Existing studies describe the high levels of stress and pressure affecting nursing home employees (uncertainty, hopelessness, excess workload, and role conflicts) [14,49], with a significant impact on their mental health [49]. High levels of anxiety, loneliness, and uncertainty are also observed among residents and family members [50].

One of the priorities in nursing homes was to reduce infections and deaths among residents, although this also led to emotional exhaustion. The Spanish government [51] has reported that between 10 March and 23 June 2020, 20,268 people died of COVID-19 in nursing homes, representing 47–50% of the total deaths in the first wave of the pandemic [51]. Similar percentages were recorded in the United Kingdom (45%), France (46%), Sweden (46%), Scotland (47%), and Northern Ireland (49%) [52]. In Spain, deaths in nursing homes were affected by factors such as [51]: (a) the highly contagious nature of SARS-CoV-2; (b) the presence of morbidity, dependency, and needs for care and direct contact among residents); (c) inadequate infrastructure for isolation and PPE shortages; (d) inadequate staff-to-resident ratios, insufficient training, and large numbers of staff on sick leave; (e) communication problems between staff, residents, and families, and information bias towards nursing homes; (f) difficulties in obtaining diagnostic tests and delivering end-of-life care, shortages of protective material, and breakdowns in the system for collecting corpses; (g) confusion between sectors and government agencies, prioritising hospitals over nursing homes and overlooking nursing homes in broader public health measures; (h) ageism towards the elderly population, discrimination against disabled people, and legal problems for failing to provide healthcare due to the collapse of the health system. Ouslander and Grabowski [53] point to a perfect storm in nursing homes, with the combination of a vulnerable population with nonspecific and atypical presentations of COVID-19, staff shortages due to viral infection, inadequate resources for and availability of rapid, accurate testing and PPE, and lack of effective treatments for COVID-19.

Despite the adversity they faced during the pandemic [32,33,34,35,36,37,38,39,40,41,42,44,45,46,47], the experiences identified in this review provide evidence of positive psychological capital (PsyCap) among nursing home professionals [34,35,36,37,38,40,41,42,45,47], as reported by other studies [54]. PsyCap is a positive individual psychological state [55] that motivates people to strive for wellbeing by developing positive emotions and an appreciation for life [56]. It is characterised by the presence of hope, optimism, resilience, and self-efficacy [56]. These psychological characteristics could explain nursing home staff’s ability to adapt to the pandemic. Hope prompts people to work to achieve their goals [57] encouraging them to find alternative solutions as new challenges arise [55], and is manifested in the form of creativity, improvisation, and confidence, as shown by this study [38,40,41,47]. The presence of satisfaction, joy, and pride [38,40,41,47] point to positive expectations of the future within nursing homes [58]. Feelings of belonging, professional dedication, duty, solidarity, and a spirit of sacrifice [34,36,41,42,47] culminating in greater group cohesion [35,40,42,45] are evidence of resilience and an ability to recover from adversity by taking on greater responsibility [59]. Finally, the ability to improvise and devise creative solutions observed in nursing homes [38,40,41,47] is associated with self-efficacy and belief in one’s own problem-solving abilities [60]. PsyCap is a resource that can help individuals tackle or alleviate the negative impacts of stress to preserve their mental health [61]. Future research could describe and analyse the factors allowing risks and hardships to be transformed into opportunities during the COVID-19 pandemic.

### Limitations

The study only includes research published up to 15 February 2021. Data on the period following the lockdown and the return to pre-pandemic dynamics are lacking. Due to the large numbers of articles published about COVID-19 and their variable indexing [62], some articles may have been overlooked in this scoping review. In this study we have used generic descriptors related to the care of elderly people living in nursing homes and their families, since the inclusion of more restrictive terms provided a very small number of articles. Finally, the aim of this scoping review was not to consider the quality of the articles published but to provide a comprehensive overview of the evidence published.

## 5. Conclusions

The COVID-19 pandemic affected the organisation of nursing homes, resulting in emotional exhaustion among professionals, residents, and family members due to the immense human and material losses experienced. Despite this adversity, creative, innovative, collaborative solutions were found to tackle the pandemic in nursing homes.

This study has important implications for human and material resource management and for the development of action plans in preparation for later waves of the pandemic. It is especially important to design protocols that clearly define the role of hospitals and primary care facilities and their support for nursing homes. It may also help to establish tools for communication between professionals and between residents and family members during lockdown.

In the future, the perspectives of residents and their family members during the pandemic will be useful to identify important aspects of their care and support in nursing homes.

## Figures and Tables

**Table 1:** Search terms and their combinations.

Combinations	English Terms	Spanish Terms
First combination	(COVID* OR pandemic* OR coronavirus infection* OR SARS*) AND (Health Personnel OR Allied Health Personnel OR Health provider OR health care workers) AND (“qualitative research” OR “mixed research”)	(COVID* O pandem* O infección coronavirus* O SARS*) Y (Personal Salud O Profesional salud O Proveedor salud O trabajadores salud) Y (“investigación cualitativa” O “investigación mixta”)
Second combination	(COVID* OR pandemic* OR coronavirus infection* OR SARS*) AND (“Nursing homes” OR “long-term facilities” OR “Health Services for the Aged” OR “Geriatric Hospitals”) AND (“Health Personnel” OR “Allied Health Personnel” OR “Health provider” OR “health care workers”) AND (Attitude OR Attitude of Health Personnel OR Perception OR experience OR Perspective OR feeling OR knowledge) AND (“qualitative research” OR “mixed research”)	(COVID* O pandem* O * infección coronavirus* O SARS*) Y (“Residencias” O “centros cuidados prolongados” O “Servicios Salud para mayores” O “Hospitales Geriátricos”) Y (“Personal salud” O “Profesional Salud” O “Proveedor salud” O “trabajadores salud”) Y (Actitud O Actitud profesional salud O Percepción O experiencia O Perspectiva O Sentimientos O conocimiento) AND (“investigación cualitativa” O “investigación mixta”)
Third combination	(COVID* OR pandemic* OR coronavirus infection* OR SARS) AND (“Nursing homes” OR “long-term facilities” OR “Health Services for the Aged” OR “Geriatric Hospitals”) AND (“Health Personnel” OR “Allied Health Personnel” OR “Health provider” OR “health care workers”) AND (“qualitative research” OR “mixed research”)	(COVID* O pandem* O * infección coronavirus* O SARS*) Y (“Residencias” O “centros cuidados prolongados” O “Servicios Salud para mayores” O “Hospitales Geriátricos”) Y (“Personal salud” O “Profesional Salud” O “Proveedor salud” O “trabajadores salud”) Y (“investigación cualitativa” O “investigación mixta”)
Fourth combination	(COVID* OR pandemic* OR coronavirus infection* OR SARS*) AND (“Nursing homes” OR “long-term facilities” OR “Health Services for the Aged” OR “Geriatric Hospitals”) AND ( “qualitative research” OR “mixed research”)	(COVID* O pandem* O * infección coronavirus* O SARS*) Y (“Residencias” O “centros cuidados prolongados” O “Servicios Salud para mayores” O “Hospitales Geriátricos”) Y (“investigación cualitativa” O “investigación mixta”)

*: The asterisk is used as a truncation symbol to search for the root of the word and retrieve any alternate endings.

**Table 2:** Variables included in the fourth search phase.

Items	Observations
Language	English, French, Portuguese, Spanish
Objectives/aims	To identify each study’s objectives and research questions.
Design	Qualitative research (phenomenology, grounded theory, etc.), mixed methods research (sequential, embedded, etc.)
Participants	Total number, distribution by type (residents, family members, healthcare professionals, social care professionals) and sex.
Setting and/or context	Nursing homes, long-term care facilities.
Sampling strategies	Purposive, convenience. Identification of end of recruitment (theoretical saturation of data, information redundancy, etc.)
Data collection tools	Interviews, observation, focus groups, questionnaires with open-ended questions. Identification of end of recruitment (theoretical saturation of data, information redundancy, etc.)
Findings	Identification of results obtained, description and classification into categories, themes, metacategories, etc.
Identification of other references	Identification of other references that may meet the inclusion criteria in the bibliography. Cross references obtained after reading document. The full screening process was applied to each additional reference.

**Table 3:** Summary of scoping review and topic area of studies.

	Deterioration of the Organisational Climate		Adapting to Adversity	
Authors	Organisational Difficulties in Responding to the New Circumstances	Experiences of Emotional Exhaustion and Negative Perceptions	Innovation and Creativity in Responses to Adversity	Acknowledging and Adapting to Shortcomings
Bergman et al., 2020 [32] ^β^	X	X	X	
Bolt et al., 2021 [44] ^β^		X	X	X
Chee, 2020 [46] *		X		
Cocuzzo et al., 2020 [33] *		X		
Cousins et al., 2021 [34] ^β^	X	X	X	X
Fearn et al., 2021 [37] ^β^	X		X	
Frahsa et al., 2020 [38] ^β,&^	X	X	X	X
Havaei et al., 2021 [39] ^β^	X	X	X	
Kabir et al., 2020 [40] ^β^	X	X	X	X
Lázaro et al., 2020 [41] ^β^	X	X	X	X
Leontjevas et al., 2020 [42] ^β^	X	X	X	X
Leskovic et al., 2020 [45] ^β^	X	X	X	X
Marshall et al., 2021 [35] ^β^	X	X	X	X
Sarabia-Cobo et al., 2020 [47] ^β^	X	X	X	X
Spilsbury et al., 2020 [36] ^β^	X		X	X
Verbeek et al., 2020 [43] ^β^	X			

Participants: * residents; ^&^ family members; ^β^ professionals.

## Supplementary Materials

The following are available online at https://www.mdpi.com/article/10.3390/ijerph181910099/s1, Table S1: Characteristics of included studies.
