# Supplementary material for: Long-Term Care Facilities and Nursing Homes during the First Wave of the COVID-19 Pandemic: A Scoping Review of the Perspectives of Professionals, Families and Residents
Source: Int J Environ Res Public Health. 2021 Sep 26;18(19):10099. doi: 10.3390/ijerph181910099 (PMC8508277; doi:10.3390/ijerph181910099)
Supplement: Supplementary file 1 [file ijerph-18-10099-s001.zip › ijerph-1376208-supplementary.pdf]

Table S1. Characteristics of included studies

Authors, Country	Objectives	Design	Participants/Qualitative data analysed	Sampling strategy	Data collection	Analysis
Bergman et al., 2020 (USA)[32]	To generate consensus guidance statements focusing on essential family caregivers and visitors.	A modified 2-step Delphi process	21 professionals (14 female, 7 male)	Not described	Likert questionnaire	Identification of topics most frequently highlighted by participants, discussion and acceptance of results by consensus.
Bolt et al., 2021 (Netherlands)[44]	To formulate practical recommendations for nursing staff on providing palliative dementia care during the COVID-19 pandemic.	A scoping review following guidelines from the Joanna Briggs Institute	23 documents: 7 (special) articles in peer-reviewed journals, 6 guides, 4 letters to editors, 2 web articles (blogs), 2 reports, a correspondence paper, and a position paper	PubMed, CINAHL, and PsycINFO databases and the Google (Scholar) search engine were used. Inclusion criteria: papers written in English or Dutch and published from December 2019 onwards. Also, grey literature. relevant letters and editorials, guides, web articles, and policy papers.	Title–abstract screening and full-text screening for study selection.	Firstly, categorisation by palliative care domain as proposed by the National Consensus Project Clinical Practice Guidelines for Quality Palliative Care. Secondly, inductive analysis and identification of main categories by consensus.
Chee, 2020 (Malaysia)[46]	To explore the lived experiences of older adults during COVID-19	Colaizzi's phenomenological method	10 residents (5 female, 5 male)	Purposive sampling	In-depth, semi-structured interviews	Colaizzi's seven stages analysis method
Cocuzzo et al., 2020 (USA)[33]	To balance the need to protect residents and the need for human touch	A letter with a first-person testimony	2 residents (1 female, 1 male)	Not described	Not described	Not described
Cousins et al., 2021 (UK)[34]	To discuss the care of people with dementia in nursing homes during the pandemic, examining the extent	A qualitative media analysis review	47 news and academic articles published during the first few months of the outbreak	Documents included based on their relevance to the enquiry	NA	Analysis, coding and synthesis

	to which care has been delivered ethically during the COVID-19 outbreak					
Fearn et al., 2021 (Australia)[37]	To gauge perceptions among volunteers befriending residents with symptoms of depression, anxiety, and loneliness	A qualitative phenomenological approach	18 volunteers (3 male, 15 female)	Convenience sampling	10 individual semi-structured interviews (2 male, 8 female)<br>8 participants interviewed as a group (1 male, 7 female)	Colaizzi's phenomenological data analysis steps
Frahsa et al., 2020 (Germany)[38]	To assess the impact of COVID-19 restrictions on PA promotion in nursing homes	An organisational-sociological approach	12 administrators (2 male, 10 female)<br>24 nursing home staff and 20 relatives	Not described	Semi-structured interviews from nursing home administrators' perspectives; open-ended surveys with nursing home staff and relatives; document collection	Reflexive thematic analysis and document analysis
Havaei et al., 2021 (Canada)[39]	To describe the leadership strategies used to manage the pandemic in one COVID-19-free long-term care facility	A mixed- methods case study	4 executive leaders	Not described	Virtual semi-structured interviews	Content analysis
Kabir et al., 2020 (Sweden)[40]	To explore experiences of the pandemic among frontline workers in the first few months	A personal report	1 nurse (1 female)	Informal approach	Individual interview	Not described
Lázaro et al., 2020 (Spain)[41]	To analyze professionals' experiences during the COVID-19 pandemic and define intervention strategies to improve emotional management and wellbeing among elderly people and professionals	A qualitative study	146 health and social care professionals (nursing assistants, nurses, doctors, social workers, psychologists, physiotherapists, nursing home directors, support staff and union representatives)		Individual and group interviews	Discourse analysis, use of NVivo 12
Leontjevas et al., 2020 (Netherlands)[42]	To gain an understanding of a) whether challenging behaviour in nursing home residents changed while COVID-19 measures were in place; b) whether practitioners' involvement in treating	A mixed methods study	Survey stage: 323 nursing home practitioners (200 psychologists, 76 geriatricians, 33 specialised nurses, 14 other professionals, e.g. occupational therapists, physiotherapists)	Not described	Survey of 323 NH practitioners and in-depth interviews with open-ended questions with 16 NH practitioners	Nonparametric analyses (survey), and content analysis (interviews)

	challenging behaviour changed; c) what can be learned from the experiences of NH staff		Qualitative study stage: 16 nursing home practitioners			
Leskovic et al., 2020 (Slovenia)[45]	To identify relationships and changes in satisfaction and burnout levels among healthcare professionals working in Slovenian nursing homes in rural areas, and to identify in-depth relationships and changes in both studies during the COVID-19 pandemic	A cross-organisational study using quantitative and qualitative methods to obtain generalised and in-depth results	Quantitative stage: 2013 (n = 556, 523 females, 33 male) and spring 2020 at the peak of the pandemic in Eastern Europe (n = 781, 744 females, 34 male)<br><br>Qualitative stage: 18 participants; 6 registered nurses, 6 nursing assistants and 6 nurse aides	Quantitative stage: sampling type not described<br><br>Qualitative stage: snowball sampling	Quantitative stage: online survey<br><br>Qualitative stage: in-depth semi-structured individual interviews	Quantitative stage: comparing quantitative and categorical variables to create a linear regression model. Comparative analysis of questionnaire results.<br><br>Qualitative stage: inductive analysis
Marshall et al., 2021 (UK)[35]	To inform more effective responses to the ongoing pandemic, and to improve understanding of how to work with nursing home staff and organisations after the pandemic has passed	Ethnographic, organisational systems approach, and qualitative interview-based study	10 registered nursing home managers	Snowball sampling	Interviews by videoconference or phone	A deductive-inductive approach, and an adapted organisational framework analysis with a focus on social ties and interdependencies between organisations and individuals
Sarabia-Cobo et al., 2020 (America & Europe)[47]	To explore the emotional impact and experiences of geriatric nurses working in nursing homes and caring for patients with COVID-19	A qualitative study using a phenomenological method	24 active registered nurses (24 female)	Snowball sampling	In-depth interviews via videoconference using a semi-structured interview guide	Content analysis using N-Vivo 8 software
Spilsbury et al., 2020 (UK)[36]	To identify issues and uncertainties relating to care and organization expressed by nursing home staff, and to understand what information would address these uncertainties and provide support in the short, medium and long term	A short report	250 nursing home staff	Not described	A self-formed, closed WhatsApp discussion group	An inductive thematic analysis
Verbeek et al., 2020 (Netherlands)[43]	To prevent and control COVID-19 infections, nursing homes across the world adopted highly restrictive measures, including	A mixed methods cross-sectional study	26 contact persons: nursing home managers (n = 16), local quality or policy officers (n =	Participants who possessed the most information on policy and local	An electronic questionnaire, telephone interviews, document analysis (i.e. local visiting	Descriptive statistics were calculated for questionnaire responses.

	bans on visitors. This study reports on initial findings on how guidelines were applied in the local context; compliance with local protocols; and the impact on wellbeing among residents, family caregivers, and staff.		8), and registered nurses (n = 2)	protocols for nursing homes or who were involved in developing local guidelines were selected	protocols), and a WhatsApp group	Data from the open-ended questions and the WhatsApp group were analyzed thematically by the research team.
